# The Role of Viral Infections in the Onset of Autoimmune Diseases

**DOI:** 10.3390/v15030782

**Published:** 2023-03-18

**Authors:** Bhargavi Sundaresan, Fatemeh Shirafkan, Kevin Ripperger, Kristin Rattay

**Affiliations:** Institute of Pharmacology, Biochemical Pharmacological Center, University of Marburg, 35043 Marburg, Germany

**Keywords:** central tolerance, peripheral tolerance, autoimmunity, molecular mimicry, bystander activation, epitope spreading, rheumatoid arthritis, multiple sclerosis, systemic lupus erythematosus, type 1 diabetes, COVID-19

## Abstract

Autoimmune diseases (AIDs) are the consequence of a breach in immune tolerance, leading to the inability to sufficiently differentiate between self and non-self. Immune reactions that are targeted towards self-antigens can ultimately lead to the destruction of the host’s cells and the development of autoimmune diseases. Although autoimmune disorders are comparatively rare, the worldwide incidence and prevalence is increasing, and they have major adverse implications for mortality and morbidity. Genetic and environmental factors are thought to be the major factors contributing to the development of autoimmunity. Viral infections are one of the environmental triggers that can lead to autoimmunity. Current research suggests that several mechanisms, such as molecular mimicry, epitope spreading, and bystander activation, can cause viral-induced autoimmunity. Here we describe the latest insights into the pathomechanisms of viral-induced autoimmune diseases and discuss recent findings on COVID-19 infections and the development of AIDs.

## 1. Introduction

A key feature of the immune system is the capability to distinguish between self and non-self. Defects in self/non-self discrimination increase the risk of infections and malignancies, while abnormal immune responses cause immunopathologies and autoimmune diseases (AIDs). Approximately 5% of the world’s population suffer from autoimmune disorders, which are lifelong and functionally diverse and are steadily increasing in Westernized societies.

AIDs result from dysregulated immune responses that can either be of extended duration, lacking the necessary downregulation, or be too vigorous and intensive, without the necessary counterbalance of immune response regulation [1,2,3]. Based on the number of targeted tissues, AIDs are categorized into organ-specific autoimmune diseases, such as type I diabetes (T1D or DMT1, diabetes mellitus type 1), multiple sclerosis (MS), inflammatory bowel disease (IBD) and myasthenia gravis and secondly, systemic autoimmune diseases, such as systemic lupus erythematosus (SLE), rheumatoid arthritis (RA) and Sjögren syndrome, in which multiple tissues or cell types can be targeted by the host’s immune system. Although the pathomechanisms of many of the autoimmune diseases are not fully understood, significant advances have been made in characterizing the deregulated immune responses, identifying the involved immune cells and cytokine signaling, identifying putative causes and in developing specific therapies [4].

Genetic predisposition is one of the contributing risk factors for several autoimmune diseases. Among these are rare monogenic autoimmune diseases in which single gene mutations or dysfunctions lead to immunopathologies. The monogenetic autoimmune disease autoimmune polyendocrinopathy syndrome type 1 (APS1) (also called autoimmune polyendocrinopathy candidiasis ectodermal dystrophy (APECED)) is a multiple organ-specific autoimmune disease in which a mutation in the autoimmune regulator (*AIRE*) gene is causative to the breach of central immune tolerance [5]. Another monogenic AID is autoimmune lymphoproliferative syndrome (ALS), which is characterized by the accumulation of polyclonal double-negative T cells (CD3^+^TCRαβ^+^CD4^−^CD8^−^) caused by mutations in Fas or Fas ligands, or in caspases downstream of Fas signaling [6]. The monogenic autoimmune disease IPEX (immunodysregulation polyendocrinopathy enteropathy X-linked syndrome) is caused by a defect in the Foxp3 gene which leads to the loss of CD4^+^CD25^+^ Tregs and peripheral tolerance [7].

The majority of autoimmune diseases are, however, not caused by a monogenic disorder, but rather have multiple genetic and non-genetic causes that play a role. Genome-wide association studies led to the identification of hundreds of loci associated with autoimmunity in rheumatoid arthritis, multiple sclerosis and inflammatory bowel disease [8,9]. Among the genetic loci, major histocompatibility complex (MHC) haplotypes are well studied and reported to show strong associations to autoimmune diseases [10,11]. Additionally, other genes such as NOD2 (Crohn’s disease), IBD5 (IBD), PTPN22 (T1D, RA, SLE and Graves’ disease), CTLA4 (T1D), IL23R and TYK2, have been implicated as AID risk factors [8,12,13,14,15,16].

Numerous environmental factors, such as pathogens, toxic chemicals and dietary influences, are known to have an impact on the immune system, and potentially function as contributing factors in the development of autoimmune conditions [17,18]. Chronic viral infections are one of the major environmental factors that are thought to be able to trigger autoimmunity. This research area has long been studied and has been previously reviewed as well [19,20,21,22,23]. The coronavirus disease 2019 (COVID-19) pandemic led to the infection of millions of people worldwide. Early on, autoantibody production and the correlation with severe COVID-19 cases was described [24]. Moreover, an increasing number of reports have accumulated in which AIDs developed after severe acute respiratory syndrome coronavirus 2 (SARS-CoV-2) infections [25]. Hence, the prospective role of viral infections as triggers for the development of autoimmune diseases is coming into focus again. Due to the complex etiologies of autoimmune diseases, suitable therapies to cure these diseases are difficult to develop. A better understanding of the involved cell types and mediators is needed in order to identify possible anchor points for future therapies. In this review, we discuss virus infections as one of the major causes of autoimmunity, focusing on the role of virus-induced inflammation and deregulated immune reactions. In this review, we present additional potential etiologic information and discuss recent publications and newly obtained data on this research topic. We summarize recent findings on rheumatoid arthritis (RA), systemic lupus erythematosus (SLE), multiple sclerosis (MS) and diabetes mellitus type 1 (DMT1), and their mechanisms of pathogenesis.

## 2. Immune Tolerance

A multitude of pathogens pose a constant threat to the health and wellbeing of humans. Clearing the infections is an indispensable task of the immune system to ensure survival. The vast diversity of the B cell and T cell receptor repertoire is essential for the recognition and removal of invading pathogens. On the one hand, the high receptor repertoire is important in order to be able to recognize and fight many different pathogens; on the other hand, the mechanisms of gene recombination and somatic hypermutation that ensure the high variation of the receptors also increase the risk of generating receptors that recognize self-antigens. Rigorous selection processes in the thymus for developing T-lymphocytes and in the fetal liver and bone marrow for B-lymphocytes remove the majority of autoreactive T and B cells before they leave the primary lymphoid organs (central tolerance) [26].

Medullary thymic epithelial cells are capable of expressing self-antigens (e.g., tissue restricted antigens (TRAs)) and present these on MHC molecules to developing thymocytes for negative selection [27,28]. Self-antigen expression in mTECs is affected by the transcriptional regulator AIRE and the transcription factor Fezf2, epigenetic modifications and yet unknown factors driving the expression in defined co-expression patterns of self-antigens, leading to a heterogeneous mosaic expression pattern of self-antigens in mTECs [29,30,31,32,33,34,35]. Additionally, thymic dendritic cells (tDCs) play an important role in the negative selection, presenting a complementary set of antigens, but also cross-presenting mTEC-derived antigens as well as peripheral, imported antigens [36,37]. Further, thymic B cells were shown to contribute to thymic negative selection [38]. Autoreactive B and T-lymphocytes are replenished from the pool of newly generated lymphocytes through clonal deletion, clonal diversion (e.g., development into regulatory T cells (Tregs)), further receptor editing (via secondary gene rearrangement) or the induction of anergy [39,40,41]. However, despite the effectiveness of central tolerance selection, the development of self-reactive lymphocytes cannot be prevented completely, and autoreactive B and T cells are known to be part of the peripheral lymphocyte pool [42,43]. Instead, different protective measures are in place that help to regulate immune responses in their duration, intensity and location. Among these, Tregs play an important role in regulating immune responses in the periphery, also referred to as peripheral tolerance [44].

Autoimmune diseases result from a loss of immunological tolerance to self-antigens. Different scenarios are described to date, in which either a breach of central tolerance selection or insufficient peripheral suppressive functions are the underlying cause [1,2,3]. A prominent example for a loss of central tolerance and the development of AID is the monogenic disorder APECED (or APS1) in which a mutation in the gene encoding AIRE leads to multiorgan autoimmunity. AIRE is a transcriptional regulator involved in the regulation of a multitude of self-peptides in medullary thymic epithelial cells (mTECs) and is known to be essential for mTEC maturation and T cell selection [45,46,47,48]. Moreover, faulty expression of individual TRAs is sufficient to drive organ-specific autoimmunity, as shown for insulin (Ins2) and the development of autoimmune diabetes. Another example is the loss of thymic expression of interphotoreceptor retinoid-binding protein (IRBP) which results in uveitis [49,50,51].

Viral infection of the thymus has been reported to alter T cell maturation and selection and proposed to lead to autoimmunity. Human and mouse roseoloviruses, members of the herpesvirus family, were shown to infect the thymus and lead to a transient depletion of CD4^+^ single positive and CD4^+^CD8^+^ double positive thymocytes [52,53]. Using neonatal roseolovirus infections in mice, researchers could show a direct link between the viral infection and the development of autoimmune gastritis. Twelve weeks after infection, the mice developed autoimmune phenotypes resulting in stomach inflammation with autoantibodies of diverse specificity and autoreactive CD4^+^ T cells [54]. Interestingly, a transient reduction in mTECs, tDCs and AIRE and TRA expression was observed after neonatal infections with roseoloviruses in mice.

Of particular importance for peripheral tolerance are CD4^+^CD25^+^FOXP3^+^ Treg (regulatory T cells). Tregs can also promote tissue repair in response to inflammatory factors released from damaged cells [55]. Tregs can develop intrathymically (natural Tregs; nTregs) out of CD4^+^ single positive thymocytes with reactivity towards self-antigens or from conventional CD4^+^ T cells in the periphery after antigen encounter (induced Tregs; iTregs) under the influence of TGF-β and IL-2 [56,57]. Numerous autoimmune conditions, including type 1 diabetes, rheumatoid arthritis, multiple sclerosis, systemic lupus erythematosus and myasthenia gravis have been linked to Treg dysfunction [58].

T and B cells express several inhibitory molecules on their surfaces, such as CTLA-4, PD-1, LAG-3, TIM3, VISTA, TIGIT, FcγRIIb and Siglec proteins. Deficiencies in these inhibitory molecules can lead to the development of autoimmune diseases due to a lack of counterbalance of the immune reaction. Blockage of these inhibitory surface molecules by therapeutic antibodies is used in anti-tumor therapies, known as checkpoint immunotherapies [43,59,60].

The development of therapies to induce or enhance immune tolerance through the interruption of effector mechanisms, restraining innate immune activation or by boosting immune response regulation has been the focus of several clinical trials [61]. Immunomodulatory B and T cell targeting, the induction of exhaustion and the regulation of costimulatory pathways are promising angles for therapies and were shown to be effective in AIDs such as T1D and anti-neutrophil cytoplasmic antibody (ANCA)-associated vasculitis [61].

## 3. Inflammation-Induced AID

A functioning immune system is essential to fight pathogen infections caused by viruses, bacteria, fungi or parasites as well as removing cancerous cells. However, prolonged proinflammatory responses to infections have been associated with the occurrence of autoimmune diseases. Proinflammatory cytokines such as type I interferon (IFN), IFN-γ (type 2 IFN), interleukins, such as IL-1β, IL-12, IL-17, and tumor necrosis factor (TNF)-α are critical mediators of immune reactions towards pathogens and several of them are implicated in the initiation of autoimmune reactions as well.

As the first line of defense, the innate immune system is particularly important in fighting pathogens. The mechanisms of the innate immune response play a crucial role in eliminating microbial infections and in repair mechanisms of damaged tissues. Further, the innate immune system initiates cytokine release and signals to initiate the adaptive immune response. Pathogen recognition by the innate immune system is accomplished through pattern recognition receptors (PRRs) that recognize pathogen-associated molecular patterns (PAMPs) in the case of infections, and damage-associated molecular patterns (DAMPs) of damaged tissues. The detection by PRRs is achieved through membrane-bound PRRs (such as toll like receptors (TLRs) and c-type lectin receptors (CLRs)) and cytoplasmic PRRs (e.g., NOD-like receptors (NLRs)). PRRs are expressed by macrophages, dendritic cells (DCs), neutrophils and epithelial cells [62]. The recognition of pathogens or damaged cells through PRR engagement triggers an intracellular signaling cascade involving NFκB-mediated transcription, inflammasome activation and caspase-1 activation, resulting in the secretion of interleukin (IL)-1β and IL-18. The inflammasome is an intracellular, multiprotein component and an essential part of the innate immune response, which was first described 21 years ago [63]. Different types of inflammasome subsets with distinct compositions are described and are engaged by different pathogens [64,65,66]. The NLR family subset is composed of NLRP1, NLRP3, NLRP6, NLRP12, and caspase activation and recruitment domains (CARD)-containing protein 4 (NLRC4). The non-NLR family subset includes absent in melanoma 2 (AIM 2) containing inflammasome complexes. Another class of PRRs are intracellular retinoic acid-inducible gene I (RIG-I)-like receptors (RLRs), including retinoic acid-inducible gene-I (RIG-I), melanoma differentiation-associated 5 (MDA5) and laboratory of genetics and physiology 2 (LGP2), which are sentinels for cytoplasmic viral RNA [67]. Triggering of RIG-1 leads to the activation of an IFN response to viral infections which was shown to aggravate lupus through enhancing IFN signaling and decreasing Tregs [68,69,70,71].

Dysregulated inflammasome activation has been implicated in the development of various autoimmune diseases, for example, rheumatoid arthritis, systemic lupus erythematosus, ankylosing spondylitis, and Sjögren syndrome, which have been well reviewed elsewhere [62,69,72]. Mostly microbial triggers have been described in activating the inflammasome; however, viruses, fungi and parasites have also been described in inflammasome activation. To date, the NLRP3 inflammasome in macrophages was shown to be activated by different viruses or viral proteins, such as the hepatitis C virus core protein [73], the viroporin of the COVID-19 causing severe acute respiratory syndrome coronavirus (SARS-CoV-2) [74], the influenza virus M2 ion channel [75], and the viroporin 2B of the encephalomyocarditis virus [74]. Further, NLRP9b, which is expressed in intestinal epithelial cells (IECs), was described as a sensor of short double-stranded RNA from rotaviruses [76]. Additionally, other virus-unspecific signals that are released upon tissue injury can lead to the activation of inflammasomes. Among these are ion efflux (cytosolic K^+^ efflux), mitochondrial dysfunction (through mitochondrial DNA (mtDNA) release) and reactive oxygen species (ROS), and possibly also metabolic dysregulation of sterol biosynthesis and glycolysis metabolism [77,78]. To what degree viral-induced or tissue damage-induced inflammasome activation is implicated in the induction of autoimmune pathologies remains to be elucidated, and further studies are needed.

## 4. Viral Mechanisms Causing AID

**Figure 1:**
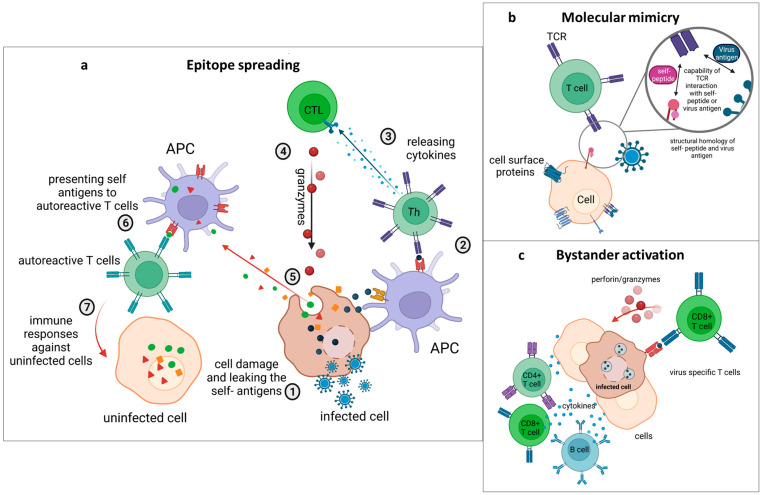
Mechanisms by which virus infections cause autoimmune diseases. (**a**) Epitope spreading. **1.** Viruses infect the host´s cells. **2.** Viral antigens are presented to T-helper cells by APCs. **3.** T-helper cells release cytokines, which can affect cytotoxic T lymphocytes (CTLs). **4.** CTLs release granzymes, which attack infected cells. **5.** Hidden self-antigens leak from damaged cells. **6.** APCs present these antigens to autoreactive T cells. **7.** Therefore, autoreactive T cells attack other uninfected cells carrying these self-antigens. (**b**) Molecular mimicry. T cell receptors (TCR) can recognize and react towards both viral antigens and self-antigens that have structural or sequential homology. (**c**) Bystander activation. Infected cells present viral antigens to virus-specific T cells. T cells identify infected cells and release cytotoxic granules, causing cell death of infected and nearby, uninfected cells. The inflammatory milieu leads to the activation of bystander cells within the tissue.

Viral infections often induce a powerful immune response that is essential for clearing the infection, but in rare circumstances, poor immune response control can result in adverse immune reactions that target the host’s antigens. Viruses, due to their capacity to activate lymphoid responses and induce inflammatory responses, were seen as a potential cause for autoimmune responses early on [23]. Chronic virus infections can induce or sustain autoimmunity through different ways, including mechanisms of molecular mimicry, bystander activation and epitope spreading (Table 1 and Figure 1).

Even though virus-induced autoimmune diseases are studied intensively and the mechanisms through which they contribute to the development of AIDs were proposed long ago, definite proof of cause and consequence is still difficult to date [79]. In the course of immune reactions towards infections, autoreactive cells can occur, but usually the infections are long cleared before the onset of autoimmunity. Additionally, autoreactive cells and autoantibodies can also be found in healthy individuals and do not necessarily lead to the development of AIDs [80,81]. Further, some viruses such as Epstein–Barr virus, which is a putative trigger of MS, can be found in almost every adult (95%) but do not lead to autoimmune reactions for everyone. Therefore, models of a primary infection, followed by latency and a secondary infection leading to the deregulated immune reaction of AIDs have been proposed [22,82].

Another factor making the identification of the causes of AIDs difficult is the statistical power of many of the epidemiologic studies. Larger cohort studies over longer periods of time, including the time before the development of autoimmunity, are necessary. Further, many of the studies use inbred murine models to mimic and study the human disease pathologies. However, findings from experimental mouse models would need to be transferred and validated in patients in order to understand the mechanistic details of the human diseases. Interestingly, sequencing technologies offer promising opportunities to study the individual human T cell repertoire in a systematic manner.

Latent virus infections in which the virus is present in the host cells but dormant without replication may contribute to the later development of autoimmune diseases. This is postulated for Epstein–Barr virus infections, which often occur early during childhood and are shown to contribute to rheumatoid arthritis (RA) later in life. A recent study described the influence of latent viral infection on the severity of symptoms using a mouse model for RA [83]. In a collagen type II-induced model of arthritis, they observed that latently infected mice showed more severe symptoms, increased Th1-responses and increased levels of age-associated B cells (ABCs).

Age-associated B cells are a subset of B cells that accumulate during immune senescence and aging [84,85] and were reviewed recently in other publications [86,87,88]. In aged individuals, higher levels of autoantibodies can be detected, and ABCs are suggested to be involved in the age-dependent accumulation of autoantibodies and the development of autoimmunity [85]. ABCs are an important component of antiviral responses and are observed to be dysregulated in viral infections such as human immunodeficiency virus (HIV) and SARS-CoV-2 [89,90]. Atypical expansion and activation of ABCs has also been associated with autoimmune pathogenesis in SLE [91]. Further, using a mouse model for a lupus-like syndrome, ABCs were found to be accumulated particularly in females [92]. Sex-dependent differences in immune responses are well described for different viral infections, efficiency of vaccinations and autoimmune diseases [93,94]. AIDs such as SLE, Graves’ disease, Hashimoto’s thyroiditis, and Sjögren syndrome have the greatest female biases, developing between seven and ten times more often in females than in males.

### 4.1. Epitope Spreading

An epitope is a sequence or site of an antigenic peptide which can be bound by the T cell receptor (TCR) or B cell receptor (BCR) of T or B lymphocytes and lead to an immune response. Antigen-specific activation of immune cells is one of the key steps during the initiation of an effective immune response of the adaptive immune system. In the course of an immune response, the epitope against which the immune response is directed can change due to two different forms of epitope spreading: (I) intramolecular spreading, in which the immunogenic epitope is a different region of the same protein; or (II) intermolecular spreading, in which the immune response is extended to an epitope of a different protein [95,96]. Usually, epitope spreading is one of the mechanisms by which the immune response against invading pathogens is broadened and intensified in order to swiftly clear the infection. However, increasing the pool of epitopes against which an immune response is initiated also leads to the increased risk of activating cross-reactive, autoreactive T or B lymphocytes [82].

The uptake of viral proteins and the processing into immunogenic antigens can lead to the exposure of peptide regions referred to as ‘cryptic’ epitopes which are otherwise hidden within the protein structure. Cryptic epitopes are unable to interact with antibodies or immune cells unless the molecule undergoes a conformational change or a conformational modification [97]. Changes in protein structure can also cause epitope spreading. Protein citrullination, which involves switching an amino acid from arginine to citrulline, is one example. Citrullination can trigger an immune response against other citrullinated proteins in addition to the original protein and its citrullinated derivative [98]. Molecular mechanisms such as endocytic processing, antigen presentation, and somatic hypermutation all contribute to epitope spreading and immune reaction amplification and are suspected to play a role in the development of autoimmune reactions [99].

Additionally, the prolonged and broadened inflammatory response can lead to the release of self-antigens from the damaged tissue and to the intermolecular epitope spreading to encompass epitopes of self-antigens, thus leading to the activation of autoreactive T or B cells (Figure 1a) [100,101]. Experimental autoimmune encephalomyelitis (EAE), a non-infectious model of MS, was the very first description of epitope spreading [82,100,102]. In this model of EAE, proteolipid protein (PLP) 139–151-specific CD4^+^ T-cell reactivity is induced initially. This is followed by an immune response towards the PLP 178–191 epitope (intramolecular epitope spreading) and the myelin basic protein (MBP) 84–104 epitope (intermolecular epitope spreading) [103]. Another example is type I diabetes, in which the B9-23 region of insulin initially sets off an autoimmune response, and the expansion of the epitope to a variety of beta cell antigens is what causes the manifestation of the disease [104]. The role of epitope spreading in the onset of autoimmune pathologies during persistent viral infections has been studied using the Theiler’s virus model [105,106,107]. Theiler’s murine encephalomyelitis virus infections can persist in glial cells, particularly astrocytes, microglial cells, oligodendrocytes or macrophages [108]. Theiler’s murine encephalomyelitis virus induces a chronic CD4^+^ T cell response to myelin. The immune response initially emerges against dominant myelin proteolipid (PLP) peptide 139–151 and is extended to the distinct and less prevalent PLP epitope 178–191 as the condition progresses [109,110,111].

Epitope spreading is also described for B cell-mediated immune responses and immune pathologies in autoimmune diseases [99]. One example is systemic lupus erythematosus (SLE), for which B cell epitope spreading in the generation of autoantibodies was described using a confetti mouse model [112].

The protective function of epitope spreading as a method to enhance the host´s defense against pathogens is also reported to play a role in the efficacy of immunotherapies [103,113]. The uptake and presentation of tumor antigens during tumor cell destruction by T cells is an important step in cancer immunotherapy, leading to the recruitment and activation of a more diverse immune response. Dendritic cells play a crucial role in antigen cross-presentation during immune responses against cancer cells [114,115]. Further, PD-1 expression by tumor-associated macrophages negatively correlates with the phagocytic activity and the induction of protective antitumor immunity. The inhibition of the PD-1/PD-L1 interaction was shown to increase the uptake of cancer cells, resulting in reduced tumor growth and prolonged survival in mice [116]. Epitope spreading in vaccination trials has been reported in patients with metastatic breast and ovarian carcinomas, malignant melanoma, prostate cancer, lung cancer, pancreatic cancer and renal cell carcinomas and was observed to a greater extent in patients that developed an immune response against vaccine components [113]. In a recent study, epitope spreading was demonstrated to increase the efficiency of the immune checkpoint inhibitor anti-programmed cell death protein-1 (PD-1) in antitumor therapies in patients with melanoma [117].

### 4.2. Molecular Mimicry

One of the mechanisms by which virus infections can lead to the development of autoimmune diseases is molecular mimicry, in which peptides from microbial or viral proteins with structural similarity to self-peptides can lead to the activation of naïve, autoreactive T or B cells (Figure 1b) [118]. Such cross-reactivity can cause the immune response to shift towards attacking self, thus leading to the development of autoimmune diseases [119].

The concept of molecular mimicry was described early by Oldstone and colleagues. They identified antibodies which respond to measles virus and herpes simplex virus (HSV-1) proteins that showed cross-reactivity to an intermediate filament protein of human cells [120]. Further, they described that the hepatitis B virus polymerase (HBVP) shares six consecutive amino acids with the encephalitogenic site of the myelin basic protein in rabbits (MBP) and that recombinant virus infection in a mouse model led to the autoimmune disease experimental autoimmune encephalomyelitis [121,122].

In contrast to other non-specific mechanisms that initiate autoimmunity, such as bystander activation and superantigens, molecular mimicry directs immune responses specifically towards a certain tissue. While numerous other components are thought to be involved in the development of autoimmunity, molecular mimicry has been implicated in the onset of autoimmune phenomena in a number of disorders, including multiple sclerosis, type 1 diabetes, myasthenia gravis, systemic lupus erythematosus, and autoimmune myocarditis [22,111,123,124,125,126]

Based on epidemiological evidence, a link between infection with Epstein–Barr virus (EBV) and MS has been suggested. Recently, a mechanism of molecular mimicry between Epstein–Barr nuclear antigen 1 (EBNA1) and host GlialCAM was reported to contribute to the development of MS. Pathogenic antibodies directed against the EBV nuclear antigen EBNA1 that cross-react with glial cell adhesion protein (GlialCAM) from the CNS were identified using human cohorts and mouse models [127].

Further, mechanisms of molecular mimicry have been proposed to cause the autoimmune phenomena observed in COVID-19 syndromes in patients with SARS-CoV-2 (severe acute respiratory syndrome coronavirus 2) infections [128,129]. Neuronal proteins of the respiratory pacemaker in the brainstem were observed to show epitope similarities with SARS-CoV-2 using in silico analysis [130]. Multiple case reports exist that describe cases of autoimmune diseases, such as multisystem inflammatory syndrome, Guillain-Barré syndrome, SLE, T1D or RA, following COVID-19 syndrome [131,132,133,134,135,136,137,138]. Further analysis and larger cohorts will be needed to monitor possible relations of SARS-CoV-2 infections with the development of autoimmune diseases.

From an evolutionary point of view, it seems plausible that pathogens need to avoid their elimination by the immune system and, therefore, need to escape immune surveillance. Evolving a mimic identity to structures of the host which is well tolerated would be one way to stay invisible to immune recognition. Ultimately however, a high degree of mimicry could lead to long-term, uncontrolled pathogen infections which might impair the host’s viability. Some viruses such as the herpesvirus make use of another way to stay unrecognized: they are well known for their capability to suppress antigen presentation in order to elude the host’s immune surveillance [139,140].

### 4.3. Bystander Activation

Another mechanism through which viruses can elicit an autoimmune response is through bystander activation. In contrast to epitope spreading and molecular mimicry, bystander activation is a mechanism by which autoimmune reactions are triggered in an antigen unspecific manner. Bystander activation is initiated by signals, including ligands for co-signaling receptors, cytokines, chemokines or extracellular vesicles containing pathogen particles, that lead to the activation of lymphocytes [141,142,143]. Thereby, a local proinflammatory environment created in an antiviral immune response can lead to the activation and expansion of autoreactive bystander T or B cells [21].

The inflammatory milieu leads to the release of further proinflammatory factors such as tumor necrosis factor (TNF), TNF-β, lymphotoxin (LT), and nitric oxide (NO), [144,145]. In the event of a viral infection, virus-specific T cells travel to the infected tissue, where they encounter viral peptides presented on MHC class I molecules on infected cells. Thereupon, CD8^+^ T cells release cytotoxic granules leading to cell death of the infected, targeted cells, potentially damaging neighboring healthy cells as well (Figure 1c) [21]. The tissue damage can lead to a leakage of self-peptides into the inflammatory milieu. Cell debris is usually cleared from the tissue through phagocytosis by macrophages. However, the presence of self-peptides in the context of an inflammatory environment also increases the risk of autoreactive T or B lymphocytes becoming activated.

The initiation or recurrence of several AIDs, including rheumatoid arthritis, systemic lupus erythematosus, type 1 diabetes, and multiple sclerosis, has been attributed to bystander activation [141,146,147]. Inflammatory cytokines produced by the innate immune response, such as IL-21 and IL-15, were shown to be able to trigger autoreactive CD8^+^ T cells in a transgenic mouse model of autoimmune diabetes [148]. In this study, the authors used a mouse model in which the glycoprotein (GP) Ag of lymphocytic choriomeningitis virus (LCMV) is expressed under the rat insulin promoter (RIP-GP). CD8^+^ T cells that express the transgenic P14 TCR (P14 cells) recognizing an antigenic peptide of LCMV GP (gp33) were activated to induce diabetes in RIP-GP mice by LCMV infection or by immunization with gp33 peptide and concomitant induction of inflammatory response by a TLR3 ligand. Further, in a similar approach, also using the RIP-GP mouse model of autoimmune diabetes, IL-7 led to the activation of autoreactive CD4^+^ T cells that can prime and expand endogenous autoreactive CD8^+^ T cells [149]. In the NOD mouse model, genetic ablation of IL21Ra abrogated the development of autoimmune diabetes [41,42,43]. However, whether Il-21 also contributed to the initial triggering of autoreactive CD8^+^ T cells was not addressed. In a murine model of autoimmune colitis, IL-6-dependent spontaneous proliferation of colitogenic CD8^+^ T cells and the induction of IL-17-producing effector CD8^+^ T cells were shown to underlie the pathogenic process [150]. Further, memory CD4^+^ T cells have been shown to undergo bystander activation and increase the autoimmune pathogenesis in mice with experimental autoimmune encephalomyelitis (EAE), a mouse model for multiple sclerosis [151,152]. Memory-like Th17 cells producing IL-17A, interferon (IFN)-γ, and GM-CSF were shown to increase the susceptibility to experimental autoimmune encephalomyelitis in an IL-1 receptor-dependent manner [152]. Furthermore, in humans, the activation of cytotoxic T lymphocytes (CTLs) via a TCR-independent NKG2D signaling pathway by IL-15 was suggested to be causing celiac disease [153].

The above-described mechanisms of epitope spreading, molecular mimicry and bystander activation are not mutually exclusive reactions through which autoimmunity can be induced. More likely, these mechanisms occur in concert with one another and in addition to other environmental triggers such as toxins, hereditary genetic predispositions, and epigenetic and nutritional influences. Ultimately, a combinatorial effect of factors favoring the development of autoimmune diseases is counteracted by regulatory factors such as central and peripheral tolerance.

## 5. Rheumatoid Arthritis

**Figure 2:**
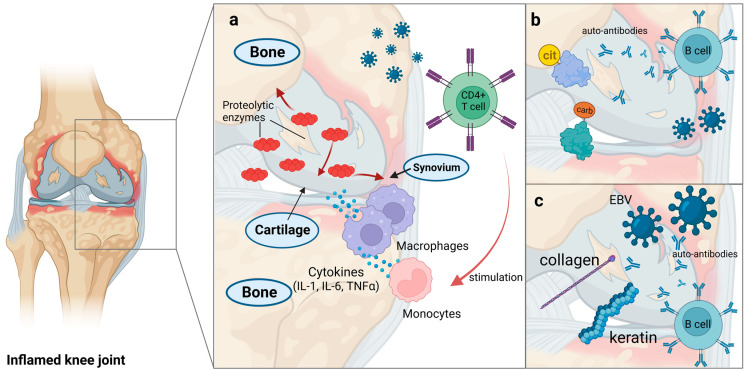
Virus-induced rheumatoid arthritis. (**a**) Synovium, bone and cartilage damage in RA. The activated CD4^+^ T cells against viruses stimulate the release of cytokines, such as IL-1, IL-6 and TNFα, from monocytes and macrophages. These cytokines lead to the increased release of proteolytic enzymes which cause damage of the synovium, cartilage and underlying bone. (**b**) Antibodies against proteins. Post-translationally changed proteins through citrullination (ACPA) and carbamylation (anti-CarP antibodies) can be identified by autoantibodies. (**c**) Molecular mimicry mechanism in RA. Antibodies reactive towards the primary epitope p107 of the EBV-encoded EBNA-1 antigen cross-react with keratin and denatured collagen.

Rheumatoid arthritis (RA) is a chronic inflammatory autoimmune condition which affects 1% of the world population [154]. Several risk factors for the development of RA are known, including genetic and epigenetic factors such as human leukocyte antigen (HLA) loci, as well as environmental factors, including tobacco smoking, obesity, low vitamin D levels and infections [155,156,157,158]. RA is characterized by a progressive damage of synovial joints with bone and cartilage destruction. The pathogenesis of RA involves several cytokines, effector cells, and signaling pathways. The degradation of the joints is initiated at the synovial membrane and is mediated by the intricate interplay of these immune modulators [159]. Lymphocyte, monocyte/macrophage and fibroblast infiltration as well as synovial hyperplasia are involved in the pathogenesis of RA [160,161,162] (Figure 2a).

**Table 1:** Summary of autoimmune diseases for which a viral cause is proposed. Listed are the respective targeted tissues or host cells, the pathology-mediating autoreactive immune cells and the proposed pathomechanisms.

Autoimmune Disease	Virus	Target Cells or Self-Peptides	Immune Cells or Cytokines	Pathomechanism	Ref.
Rheumatoid Arthritis	HTLV	Synovial cells	IL1, IL6, TNF-α	Autonomous proliferation of synovial cells and inflammation	[163,164,165]
	EBV	Collagen and keratin	Autoreactive T cells	Molecular mimicry	[166,167,168]
Multiple Sclerosis	Herpes simplex virus (HSV1, HSV2)	Peripheral sensory nerves, sensory ganglia in CNS	Cytotoxic T lymphocyte, IL-6	Molecular mimicry of HSV-1 glycoprotein gB epitope and a brain-specific factor	[169,170]
	Epstein–Barr Virus (EBV)	CNS, myelin basic protein (MBP), anoctamin 2, glial cell adhesion molecule (GlialCAM)	CD4^+^ and CD8^+^ T cells	Antibodies against EBV antigens viral capsid antigen (VCA), Epstein–Barr nuclear antigen 1 (EBNA1), and early antigen (EA), Epstein–Barr latent membrane protein 1 (LMP1), molecular mimicry	[127,171,172,173,174,175,176]
	Human Herpesvirus 6 (HHV-6)	Oligodendrocyte, MBP	T cells	Molecular mimicry of virus peptide U24 with MBP	[177,178,179]
	Varicella-Zoster Virus (VZV)	Ganglia in CNS, peripheral blood mononuclear cells (PBMCs)	CD4^+^ and CD8^+^ T cells	-	[180]
	Human Endogenous Retroviruses (HERVs)	CNS in white matter lesion, (PBMCs)	TNF-α	Induction of free radicals, ER stress	[169,170,181,182]
Systemic Lupus Erythematosus	EBV	B- and epithelial cells; autoantibodies: SmB and Ro60	EBV-specific T cells	Molecular mimicry	[183,184,185]
Diabetes Mellitus Type 1	Coxsackie B4	GAD65 in the beta-cells of pancreas	IFN-γ/type-1-IFN, IL-4	Molecular mimicry	[186]
	Rubella	GAD65/67 of the pancreatic islets	CD4^+^ and CD8^+^ T cells	Molecular mimicry	[187,188]
	Rotavirus	Tyrosine phosphatase IA-2	Type I IFN	Molecular mimicry and Bystander activation	[189,190]
	Cytomegalovirus	GAD65	GAD65 specific T-cells	Molecular mimicry	[191]

Rheumatoid factor (RF), which consists of autoantibodies against the Fc portion of IgG, antibodies against post-translational modifications such as citrullination (anti-citrullinated peptide antibodies; ACPA) and carbamylation (anti-CarP antibodies) are the types of autoantibodies often occurring in patients with RA (Figure 2b) [192,193]. Further, proteins involved in inflammatory pathways are associated with autoantibody formation in RA, including CTLA4 and PTPN22 (protein tyrosine phosphatase nonreceptor type 22), as well as PAD (peptidylarginine-deiminase) enzymes that regulates citrullination [194]. Following citrullination, acetylation or carbamylation, antigenic peptides are bound to MHC molecules on antigen-presenting cells and presented towards T cells. These in turn stimulate B cells and lead to the production of autoantibodies against the modified antigens [157]. Additionally, inflammatory cytokines including IL-1, IL-6, and tumor necrosis factor (TNF)-α are produced by monocytes and macrophages upon CD4^+^ T cells stimulation. These cytokines increase the production of proteolytic enzymes, which lead to the damage of the synovium, cartilage, and underlying bone [74]. The synovium is composed of macrophage-like synoviocytes, fibroblast-like synoviocytes (FLSs) in an intimal lining and a sublining of fibroblasts, immune cells, blood vessels and adipocytes. Macrophage-like synoviocytes are the source of pro-inflammatory cytokines TNFα, IL-1, and IL-6. The destruction of the cartilage is caused by FLSs releasing matrix metalloproteinases such as collagenases and stromelysins [195,196,197].

Viral infections have long been associated with the development of rheumatoid arthritis. RA-like polyarthritis was shown to be diagnosed clinically shortly after exposure to viruses, such as rubella virus, HTLV-1, parvovirus B19, chikungunya virus, hepatitis-B virus (HBV) and hepatitis-C virus [198,199,200,201,202]. Further, microbial infections such as *Porphyromonas gingivalis*, which causes periodontal diseases, and *Escherichia coli* have been associated with the induction of RA [119]. According to epidemiology analysis, HTLV-1 infection elevates the risk of RA and Sjögren syndrome amongst rheumatic disorders [19,163,164]. Human T-cell lymphotropic virus type I (HTLV-I) is a well-known retrovirus with the ability to promote cell proliferation in human synovial cells. HTLV-I cis activates genes in synoviocytes, primarily in synovial fibroblasts. The viral infection leads to the activation of pro-oncogenic genes and genes encoding pro-inflammatory cytokines such as IL-1, IL-6, and TNF-α, which leads to synovial hyperplasia and inflammation [165].

Further, Epstein–Barr virus infection has been associated with the induction of RA in patients and in humanized mouse models which developed arthritis-like symptoms [166]. Several serological studies have discovered that RA patients have greater levels of antibodies against EBV antigens and greater antibody titers compared with control specimens [203,204,205]. However, other serological studies observed no such correlation and instead reported an inverted correlation with low levels of antibodies against viral antigens in patients with ACPA-positive RA [206,207]. In addition to serological evidence, viral DNA has also been found in the synovium and the bone marrow of patients with RA [208]. As discussed above, the loss of tolerance in autoimmune diseases is significantly influenced by molecular mimicry. Molecular mimicry followed by epitope spreading are shown to be the major mechanisms through which EBV elicits the pathogenesis of RA [167]. Antibodies against the primary epitope p107 of the EBV-encoded EBNA-1 antigen were found to be cross-reactive to keratin and denatured collagen (Figure 2c) [168,209]. Further, Il-2 reactive antibodies were described and implicated in the altered regulation of Tregs in RA [167].

The Parvoviridae family of single-stranded DNA viruses includes parvovirus B19 which is one of the major viruses associated with RA. Arthritic symptoms appear in B19 viral infected patients, corresponding with the development of B19-specific IgM. Hence, the pathogenesis of B19-associated arthritis is thought to be fueled by virus-specific antibodies and immune complex assemblies [210].

## 6. Systemic Lupus Erythematosus

Systemic lupus erythematosus (SLE) is a systemic autoimmune condition with intervals of remission and recurrence. SLE can affect a number of organs, including the skin, joints, kidneys, renal glomeruli, lung, gastrointestinal tract and the central nervous system. Among the clinical characteristics of SLE are thrombocytopenia, hemolytic anemia, skin rash, arthritis and glomerulonephritis [211]. The humoral and cellular elements of the innate and adaptive immune response can be dysfunctional in SLE patients [212]. Several characteristics, such as anomalies in B cell tolerance and aberrant generation of numerous autoantibodies, together with impaired apoptotic cell clearance and increased type I Interferon response, immunocomplex formation and deposition are important pathophysiologic features of SLE which lead to progressive organ damage [213,214,215].

Serum autoantibodies to a wide range of antigens, such as DNA, histone and nuclear proteins, basement membrane proteins, red blood cells and platelets, are used to diagnose SLE immunologically [216,217]. Double-stranded DNA (dsDNA) and the Smith (Sm) antigen of the U-1 small nuclear ribonucleoprotein complex are the two main nuclear antigens that are regarded as diagnostic for SLE [218]. Among the environmental factors that are associated with SLE are ultraviolet light, exposure to pollution, smoking, a lack of vitamin D, viruses and microbes [219,220].

When compared with healthy people, skin lesions from SLE patients had a higher degree of immune infiltration of γδ T cells [221]. The activation of T cells plays an important role in the pathogenesis of SLE by sequestering proinflammatory cytokines, which activate B cells and thus stimulate antibody secretion [222]. The etiology of SLE has also identified the innate immune system as a major factor. Numerous abnormalities have been found in the activation status and secretory abilities of circulating and tissue-infiltrating macrophages in SLE patients and animal models. Such anomalies may be connected to SLE’s autoantibody synthesis and T cell function dysregulation. A function for macrophages in the pathogenesis of SLE was first hypothesized in response to the finding that SLE macrophages lacked the capacity to clear apoptotic cell debris, extending the time during which possible auto-antigens were exposed to the adaptive immune cells [223,224]. Further, myeloid cells obtained from SLE patients and healthy controls exhibit different gene expression profiles, revealing variations in genes that are critical for macrophage activation and polarization, with STAT1 and SOCS3 for M1 being elevated, and STAT3, STAT6, and CD163 for M2 being lowered [225]. Recently, Toll-like receptor (TLR7) signaling was demonstrated to play an important role in the development of lupus [226]. TLR7 senses viral RNA and binds to guanosine. Mutant TLR7 variants with increased sensitivity were identified in patients with SLE. Introduction of the TLR7 mutation in mice led to autoimmune reactions.

Certain viruses such as EBV and human endogenous retroviruses (HERV), including HRES-1, ERV-3, HERV-E 4-1, HERV-K10, and HERV-K18, are among the viruses that are thought to play a role in the etiology of SLE [227]. Patients with SLE have been found to have an increased prevalence of anti-EBV-humoral responses targeting the nuclear (EBNA), viral capsid (VCA), and early antigens (EA) of the virus. Antibodies specific for EBNA1 can cross-react with dsDNA, suggesting that EBV infection could induce an autoimmune response through mechanisms of molecular mimicry [184]. EBV might be causative to the autoimmune responses in some SLE patients. Moreover, SLE patients exhibit both an aberrant viral latency period and dysregulated anti-EBV response [228,229]. EBV infection of B cells, which is usually cleared by CD8^+^ T cells, has been proposed as a driver of the autoimmune reaction. In the case of latently infected memory B cells, antigen-driven differentiation can lead to the production of autoantibodies [185,230,231]. Different EBV antigens may imitate SLE antigens or other essential immune-regulatory elements structurally, molecularly, or functionally. The ribonuclear protein Sm antigen or the Ro self-protein are autoantigens associated with SLE, and the EBNA-1 antigen has sections with significant resemblance to these autoantigen sequences, such as PPPGRRP or PPPGMRPP sequences [183]. Thus, through a mechanism of molecular mimicry, the immune response against EBV nuclear antigens, which shows cross-reactivity with autoantigens, may result in SLE-specific autoantibodies. In fact, anti-Ro autoantibodies are the primary antibodies found during the preclinical stage of SLE [232,233]. In addition, retroviruses, parvovirus B19, and CMV are reported to be involved in the development of SLE. It is still unclear whether these infections precede, coincide with or develop after the commencement of SLE. Notably, the CMV protein US31 was reported to be involved in the pathophysiology and progression of SLE by causing inflammation in monocytes and macrophages through NF-κB2 activation [234]. There are also reports of SLE cases that developed after severe viral infections such as Dengue fever virus [235].

## 7. Multiple Sclerosis

**Figure 3:**
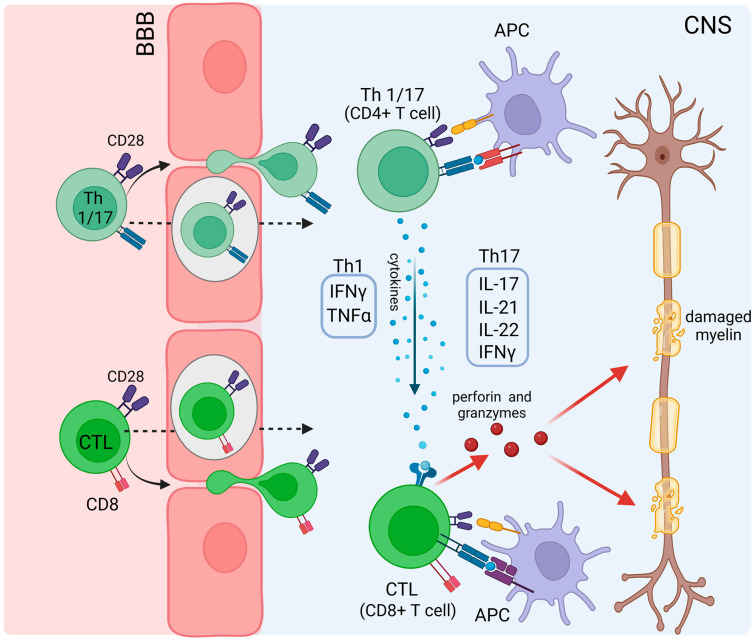
Demyelination in MS. CD4^+^ T cells (T helper 1/17 cells) and CTLs can pass through the blood–brain barrier (BBB) by endocytosis or diapedesis. Self-antigens can be presented by APCs to CTLs, Th1 or Th17 cells. Th1 cells release cytokines such as IFNγ and TNFα, whereas Th17 cells release IL-17, IL-21, IL-22 and IFNγ cytokines. These cytokines affect CTLs and increase granzyme and perforin production and release which leads to myelin destruction.

Multiple sclerosis (MS) is one of the common organ-specific autoimmune disorders of the CNS which is defined by myelin and axonal damage, and cerebral atrophy symptoms that result in permanent disabilities in patients [236,237]. Both genetic and environmental factors such as infections, nutrition, smoking and vitamin D levels, have been described in the etiology of MS [236,238,239]. Genome-wide association studies have identified >200 genetic risk variants for MS. Among these, an increased risk is associated with the HLA-DRB1*15:01 allele [240]. Demyelination is one of the characteristic types of damage in the CNS which result in MS pathology, and it can be triggered by T cell activation against myelin antigens (Figure 3) [241]. Particularly CD4^+^ T-cells, namely IFNγ-producing Th1 (T helper) cells and IL-17-producing Th17 cells and CD8^+^ T cells play central roles in MS pathogenesis and are enriched in active MS lesions in the CNS [242]. Moreover, to date, γδ T cells, innate lymphoid cells (ILCs), monocytes, macrophages and dendritic cells are known to be involved in the pathogenesis and different levels of disease severity and are comprehensively reviewed elsewhere [241]. Th1 cells release TNF and IFN-γ, whereas Th17 cells releases cytokines such as IL-17, IL-21, IL-22 and IFN-γ. These cytokines function as mediators and activate CTLs which release perforin and granzymes that result in CNS damage [243,244,245].

The blood–brain barrier (BBB) of the CNS serves as physical and functional barrier with complex tight junctions between endothelial cells. However, even at steady-state, T cells are known to pass the BBB into the CNS and be involved in the immune surveillance of the CNS [246]. In the context of MS and systemic inflammation, the permeability of the BBB is increased, promoting neuroinflammation [247]. IL-17 secreted by Th17 cells was shown to mediate neutrophil recruitment which participate in the disruption of the BBB during inflammation [248].

Myelin proteins are the most prominent autoimmune targets in MS patients, including myelin oligodendrocyte glycoprotein (MOG), myelin basic protein (MBP), and proteolipid protein (PLP), as well as lipids, such as sulfatide, sphingomyelin, and glycans (e.g., Glc(α1,4)Glc(α)) [249,250]. In addition, axo-glial proteins, such as neurofascin and contactin-2, are additional important molecular targets in MS. These antigens can be presented by APCs to naive T cells and activate autoreactive T cells. Antigen presentation can occur in secondary lymphoid organs and inside the CNS itself. The presence of myelin antigens presented by APCs and myelin-specific T cells in secondary lymphoid organs, such as spleen and lymph nodes, was demonstrated in a model of monkey experimental autoimmune encephalomyelitis (EAE) and in multiple sclerosis patients [251]. The activated myelin-specific CD4^+^ T cells differentiate into Th1 and Th17 cells, which can migrate through the BBB into the CNS [252,253]. Furthermore, migratory and tissue-resident macrophages are able to activate autoreactive T cells. For instance, microglia, being tissue-resident macrophages, upregulate MHCII, CD40, CD80 and CD86 upon inflammation and can induce T cell responses [254]. Macrophages certainly contribute to the inflammatory setting in MS; however, their precise role in the induction of MS pathogenesis is controversially discussed [241]. Based on the therapeutic effects of anti-CD20 therapies in MS patients, a role of B cell-mediated pathology is assumed [255,256]. B cells are thought to be able to trigger new MS relapses through their role in antigen-presentation, T-cell activation and cytokine production [257]. Additionally, MS patients seem to show defects in peripheral tolerance mechanisms leading to the development of autoreactive B cells [258,259]. Moreover, a study using an EAE mouse model showed that anti-MOG Ab triggered activation and expansion of peripheral MOG-specific T cells, which initiated an encephalitogenic immune response by targeting endogenous CNS antigens [260].

Among the environmental risk factors, viruses, including several herpes viruses, such as the gamma-herpes viruses, EBV and human herpesvirus 6 (HHV-6), but also paramyxoviruses (measles virus), varicella-zoster virus (VZV) and human endogenous retroviruses (HERVs) were proposed to be involved in MS pathogenesis (Table 1) [169,170,181,182]. To date, the evidence for EBV and HHV-6 as viral triggers for MS has been accumulating. However, reports on other viruses and their involvement in the development of MS remain scarce so far. VZV infection was reported to be associated with a higher risk of developing MS [180].

Several studies demonstrated an association between HHV-6 and MS either based on DNA detection in MS lesions or on increased antiviral antibody titers in MS patients [170,261,262]. However, another study did not confirm a correlation of HHV-6 DNA detection and MS pathogenesis [263]. It is noteworthy that a sequence homology and cross-reactivity between the HHV-6 protein U24 and myelin basic protein was observed, arguing for a mechanism of molecular mimicry in HHV-6-mediated MS development [177,178,179]. In a study using a model of EAE in marmosets, nonhuman primates, the authors could demonstrate that intranasal infection with HHV-6 led to accelerated EAE compared with uninfected animals [264].

The strongest evidence for virus-induced MS is documented for EBV. Several different mechanisms have been proposed for how EBV infection can trigger MS, including molecular mimicry and altered immune responses that lead to persistent EBV infections [171]. EBV can persistently infect B cells lifelong, from where the virus can intermittently reactivate. EBV-infected B cells can take up EBV antigens and present these antigens on MHC class II molecules to CD4^+^ T cells [265]. Latent EBV infection was shown to promote somatic hypermutation of BCRs, potentially generating myelin autoantigen-recognizing BCRs that could take up antigens from the CNS and stimulate autoreactive CD4^+^ T cells [266,267]. MS patients show elevated serum levels of EBV-specific antibody titers against viral capsid antigen (VCA), Epstein–Barr nuclear antigen 1 (EBNA1) and early antigen (EA) [268,269,270,271,272,273,274]. Humoral and cellular immune responses towards EBNA1 are shown to strongly correlate with MS onset [275]. Further, EBV relapses are reported to be associated with defective CD8^+^ T cell-mediated control of EBV reactivation [276]. Latent and lytic EBV transcripts were detected in MS patients’ brains compared with postmortem brain tissue of non-MS infected brain controls [277]. Antibodies to EBNA1 have been reported to be cross-reactive to anoctamin 2, myelin basic protein and glial cell adhesion molecule (GlialCAM) [127,172,278]. Additionally, antibodies against Epstein–Barr latent membrane protein 1 (LMP1) were shown to be cross-reactive towards MBP [176]. Further, EBV-induced expression of αB-crystallin in B cells was reported to lead to the activation of αB-crystallin-specific Th cells, providing another mechanism by which EBV can induce myelin-targeted inflammation [174,175]. Moreover, MBP presentation by oligodendrocytes and cross-presentation by dendritic cells was described using a mouse EAE model. The direct recognition of the oligodendrocyte-derived antigen led to the activation of naive and effector CD8^+^ T cells ex vivo [279].

Compelling evidence for EBV-induced development of MS was obtained by a longitudinal large-scale study that followed individuals starting before EBV infection until after the onset of MS. They could show that the risk for MS increased more than 30-fold after EBV infection. Serum samples of 10 million individuals in the US military were analyzed, including 810 individuals who developed MS compared with 1577 controls. The study could show that EBV infection precedes both the first neurological symptoms and early signs of neuroaxonal damage during the preclinical phase of MS [280].

## 8. Diabetes Mellitus Type 1

Type 1 diabetes (T1D or DMT1) is a chronic autoimmune disease resulting in the irreversible destruction of insulin-producing β-cells of the pancreas through autoreactive T cells and hyperglycemia [281,282]. Different genes were identified as playing a role in the genetic predisposition and as risk factors for T1D. Human leukocyte antigen (HLA) class II haplotypes DR3 and DR4-DQ-alleles were shown to be linked to the development of T1D [10]. Further, PTPN22, CTLA4, IFIH1, and IL2RA are genes that were identified as genetic risk factors [15,16,283,284]. Among the environmental risk factors, virus infections, particularly those of the gut, have been identified as risk factors for the development of T1D. Several viruses were reported to be associated with the development of T1D in humans, e.g., Coxsackievirus B, Rubella, Mumps virus, Cytomegalovirus, Epstein–Barr virus, and varicella-zoster virus (Table 1) [285]. Further, T1D and other autoimmune diseases are reported to show season-dependent variations in their incidence [286,287].

Coxsackievirus B (CVB) is one of the major enteroviruses that can be found in T1D patients [288,289]. CVB RNA sequences were found in the peripheral blood from patients at the onset of the disease or during the course of T1D [290]. Enterovirus infection was found to be two times more frequent in siblings who later developed T1D than in the control group of siblings who were nondiabetic [291]. The infection with enteroviruses starts from the gastrointestinal or respiratory system and spreads to other tissues or organs [292]. Enteroviruses are linked to a wide range of clinical manifestations such as mild febrile illness, respiratory infections, acute gastroenteritis, aseptic meningitis, encephalitis and neonatal sepsis-like syndrome [293]. CVB binds to a coxsackie and adenovirus receptor (CAR) and decay accelerating factor (DAF/CD55) on the surface of the target cells, which are mainly expressed in insulin-secretory granules [294,295]. A specific isoform of the CAR was found to be abundant in human β-cells and is localized mainly in insulin secretory granules, which might contribute to the susceptibility of human β-cells to enteroviral infection. Using T1D patient pancreatic tissue samples, the coxsackie virus B4 infection of pancreatic islet cells was shown to be specific to beta-cells, with a nondestructive inflammation mediated through natural killer cells (NK cells). Additionally, a reduced secretion of insulin in response to glucose was shown. Extracted viruses from positive islets were able to infect healthy tissue from healthy donors [296].

Persistent CVB infection rather than acute and lytic enterovirus infection was reported to lead to the induction of structural and functional changes in pancreatic and immune cells that lead to the development of autoimmunity against pancreatic islets [297,298,299,300]. Further, persistent enterovirus infection was found in 75% of gut biopsies from T1D patients (*n* = 12 type 1 diabetic patients and *n* = 10 control subjects) [301]. CVB can persist in pancreatic ductal cells, which leads to structural or functional alterations of these cells [302,303,304]. Additionally, CVB was shown to be able to persist in the intestine, blood cells and thymus in vitro and ex vivo [305,306,307,308]. These extra-pancreatic persistent infections might serve as a reservoir for the reinfection of the pancreas. Additionally, persisting infections lead to an upregulation of type-1-interfron signaling pathway components and HLA-A, -B and -C, as identified by mass spectrometry analysis on ex vivo infected primary human islet cells [309]. IFN-α was shown to mediate overexpression of HLA class I molecules, increase the expression of markers for ER (endoplasmic reticulum) stress, and lead to impaired insulin secretion and apoptosis of pancreatic β-cells [310]. The induced chronic inflammatory response promotes the recruitment and activation of pre-existing autoreactive T cells and leads to the destruction of β-cells. Ultimately, β-cells, macrophages and T cells are thought to interact in the inflammation and destruction of the tissue. Activated mononuclear cells produce cytokines (IFN-γ, IL-1β, and TNF-α) which trigger the release of chemokines and stimulatory cytokines by β-cells. Following the death of β-cells, neo-antigens are presented and can attract further mononuclear cells, leading to an amplification of the inflammation which can result in insulitis [311].

In the context of diabetes, the different mechanisms of molecular mimicry, epitope spreading and bystander activation have been discussed controversially for several viruses [124,312]. The 2C non-structural protein of CVB was proposed to induce autoimmune reactions through molecular mimicry and similarities to the glutamic acid decarboxylase 65 enzyme (GAD65), which is expressed in pancreatic β-cells [186,313,314]. Opposing studies on the presence of cross-reactive antibodies in T1D patients in which they were reported but could not be verified in other studies question the molecular mimicry mechanism for GAD65 and CVB [314,315,316,317]. Similarly, rotavirus was proposed to lead to autoimmune reactions by molecular mimicry between islet autoantigens GAD, tyrosine phosphatase IA-2 (IA-2) and the viral protein RV VP7 [189,190]. However, this association could not be confirmed in other studies [318,319]. Cross-reactivity was also described for glutamic acid decarboxylase (65 and 67) proteins and rubella virus [187]. Epitope spreading for Ins2 was shown to play a role in the pathogenesis of T1D in autoimmune diabetes of nonobese diabetic (NOD) mice [104]. Mice of the NOD model develop type 1 diabetes due to HLA variants and genetic predisposition without immunization [320]. Bystander activation of CD8^+^ T cells through IL-15- and IL-21 might be another mechanism through which the autoimmune reaction is established in T1D [148,321,322].

## 9. SARS-CoV-2 and AIDs

The coronavirus disease 2019 (COVID-19) pandemic affected millions of people worldwide. In a relatively short period of time, many people became infected with the severe acute respiratory syndrome coronavirus 2 (SARS-CoV-2) which causes COVID-19. Since then, in a growing number of case reports, the occurrence of autoimmune diseases after COVID-19 syndrome is reported [25]. The large amount of data recorded during the pandemic and the huge number of patients allow for large cohort studies to address the link between COVID-19 and the onset of AIDs. Regarding the underlying mechanism through which SARS-CoV-2 can lead to AIDs, various characteristics of COVID-19 are similar to those in autoimmune diseases. The overactivation of mature natural killer cells and CD8^+^ T cells and the dysregulation of B cells, T cells and inflammatory cytokines are immune phenotypes that could lead to the development of autoimmune reactions [90,323]. TNF-α, IL-1 and IL-6 levels, and leukocyte and neutrophil numbers were found to be elevated, whereas lymphocyte, monocyte, eosinophil and basophil counts were lower in patients with COVID-19 [324]. Bystander activation of autoreactive B cells in patients with COVID-19 was described in a small study of 23 patients [24].

The incidence of multisystem inflammatory syndrome in children (MIS-C, or Kawasaki-like disease) has been observed to increase during the COVID-19 pandemic [132,133]. Some studies reported the development of Guillain–Barré syndrome after a SARS-CoV-2 infection [131,134,135]. Cases of SLE induced by COVID-19 have been reported [136,137]. A study showed that the SARS-CoV-2 virus is able to infect pancreatic β-cells. Pancreatic autopsy tissues from COVID-19 patients were analyzed and an infiltration of SARS-CoV-2 into β-cells was detected. Additionally, it was shown that in 11 patients, ACE2, TMPRSS and other receptors such as DPP4, HMBG1 and NRP1 were expressed [325]. The nephrotic cell death, immune cell infiltration and infection of β-cells could be reasons for metabolic dysregulation in COVID-19 patients [326,327,328].

Importantly, in a large retrospective cohort study using the TriNetX database, COVID-19 patients with a positive PCR test were compared with a PCR-negative cohort (*n* = 887,455 per cohort) and were propensity score-matched for age, sex, race, adverse socioeconomic status, lifestyle-related variables, and comorbidities [138]. The authors identified a significantly higher risk within the first 6 months after COVID-19 for the development of rheumatoid arthritis, ankylosing spondylitis, systemic lupus erythematosus, dermatopolymyositis, systemic sclerosis, Sjögren syndrome, mixed connective tissue disease, Behçet’s disease, polymyalgia rheumatica, vasculitis, psoriasis, inflammatory bowel disease, celiac disease and type 1 diabetes mellitus. Hence, an awareness of the link between COVID-19 and the risk of developing AIDs is important in order to recognize autoimmune manifestations in patients and provide early treatment. Further studies will be needed to replicate the results obtained so far and to analyze parameters such as increased vaccination rates of the population.

It is noteworthy that in vitro, ex vivo, organoid models and animal models have been useful in studying the COVID-19 pathology and might be helpful in addressing the connection between SARS-CoV-2 infections and the development of AIDs as well [329,330,331]. As mice are insusceptible to SARS-CoV-2 infections due to amino acid substitutions in ACE2, the transgenic human ACE2 (hACE2)-expressing mouse model K18-hACE2 has emerged as very useful [332,333,334,335].

## 10. Outlook

Pathophysiological abnormalities and frequently overt disease emerge from the extreme ends of the range between a lack of reaction, as in immunodeficiency, and an improper, overwhelming response, as in the case of autoimmune disease. Generally, two primary classifications have been used to classify lymphocyte tolerance mechanisms. Central tolerance is based on developing T or B cells in the thymus or bone marrow, respectively. It may not come as a surprise that an aggressive immune response to an invasive disease could impair this control and cause autoimmunity. On the other hand, peripheral tolerance acts on mature T or B cells that are passing through the blood, lymph, and secondary lymphoid organs upon leaving the primary lymphoid organs. Nevertheless, there is a multitude of causes for the development of autoimmune conditions. Immunological, hormonal, and genetic predispositions and environmental factors such as infections play a role in the pathogenesis of AIDs. There is growing evidence that environmental factors are involved in the development of autoimmunity, as shown by epidemiological studies, experimental evidence and animal models. As discussed in this review, viral infections are considered a major environmental trigger causing AIDs. Viruses are shown to trigger autoimmunity through several mechanisms. Chronic viral infections are also known to sustain inflammation for a longer time and thereby lead to autoimmunity. Some of the discussed mechanisms through which viruses trigger autoimmunity are molecular mimicry, epitope spreading, bystander activation, and/or immortalization of infected B cells. In order to understand how viral infections and host autoimmune responses interact and to provide a precise molecular description of how a viral infection can lead to autoimmune conditions, additional clinical and molecular research is required. More population-based epidemiology studies on autoimmune diseases with long-term data collection from the period before the disease manifests to the development of symptoms and disease are needed. Such studies allow the assessment of trends, risk factors, coexisting morbidities, the costs of disease, while identifying differences among population subgroups and determining the prevalence of under-researched autoimmune diseases, such as celiac disease. During the COVID-19 pandemic, large data set acquisition and worldwide patient cohorts were collected that can serve as a valuable resource to monitor the development of AIDs and the connection with SARS-CoV-2 infection.

## Data Availability

Not applicable.

## Abbreviations

AIDs	Autoimmune diseases
AIRE	Autoimmune regulator
APCs	Antigen presenting cells
BBB	Blood brain barrier
CMV	Cytomegalovirus
CNS	Central nervous system
cTECs	Cortical thymic epithelial cells
CTL	Cytotoxic T lymphocytes
DAMPs	Damage-associated molecular patterns
DCs	Dendritic cells
DMT1	Diabetes mellitus type 1
EBV	Epstein–Barr virus
ER	Endoplasmic reticulum
Fezf2	Fez family zinc finger protein 2
HERV	Human endogenous retrovirus
HHV	Human herpesvirus
HIV	Human immunodeficiency virus
HSV	Herpes simplex virus
IFN	Interferon
IL	Interleukin
MHC	Major histocompatibility complex
MS	Multiple sclerosis
mTECs	Medullary thymic epithelial cells
NF-kβ	Nuclear factor kappa-β
NK	Natural killer
PAMPs	Pathogen associated molecular patterns
RA	Rheumatoid arthritis
SLE	Systemic lupus erythematosus
T1D	Type 1 diabetes
TCR	T-cell receptor
TECs	Thymic epithelial cells
TF	Transcription factor
TNF	Tumor necrosis factor
TRAs	Tissue restricted antigens
Tregs	Regulatory T cells
VZV	Varicella-zoster virus
